# Assessment of dead-space ventilation in patients with acute respiratory distress syndrome: a prospective observational study

**DOI:** 10.1186/s13054-016-1311-8

**Published:** 2016-05-05

**Authors:** Jonne Doorduin, Joeke L. Nollet, Manon P. A. J. Vugts, Lisanne H. Roesthuis, Ferdi Akankan, Johannes G. van der Hoeven, Hieronymus W. H. van Hees, Leo M. A. Heunks

**Affiliations:** Department of Critical Care Medicine, Radboud University Medical Center, Geert Grooteplein Zuid 10, 6525 GA Nijmegen, The Netherlands; Department of Pulmonary Diseases, Radboud University Medical Center, Geert Grooteplein Zuid 10, 6525 GA Nijmegen, The Netherlands

**Keywords:** Acute respiratory distress syndrome, Dead space, Douglas bag, Indirect calorimetry, Volumetric capnography

## Abstract

**Background:**

Physiological dead space (V_D_/V_T_) represents the fraction of ventilation not participating in gas exchange. In patients with acute respiratory distress syndrome (ARDS), V_D_/V_T_ has prognostic value and can be used to guide ventilator settings. However, V_D_/V_T_ is rarely calculated in clinical practice, because its measurement is perceived as challenging. Recently, a novel technique to calculate partial pressure of carbon dioxide in alveolar air (PACO_2_) using volumetric capnography (VCap) was validated. The purpose of the present study was to evaluate how VCap and other available techniques to measure PACO_2_ and partial pressure of carbon dioxide in mixed expired air (PeCO_2_) affect calculated V_D_/V_T_.

**Methods:**

In a prospective, observational study, 15 post-cardiac surgery patients and 15 patients with ARDS were included. PACO_2_ was measured using VCap to calculate Bohr dead space or substituted with partial pressure of carbon dioxide in arterial blood (PaCO_2_) to calculate the Enghoff modification. PeCO_2_ was measured in expired air using three techniques: Douglas bag (DBag), indirect calorimetry (InCal), and VCap. Subsequently, V_D_/V_T_ was calculated using four methods: Enghoff-DBag, Enghoff-InCal, Enghoff-VCap, and Bohr-VCap.

**Results:**

PaCO_2_ was higher than PACO_2_, particularly in patients with ARDS (post-cardiac surgery PACO_2_ = 4.3 ± 0.6 kPa vs. PaCO_2_ = 5.2 ± 0.5 kPa, *P* < 0.05; ARDS PACO_2_ = 3.9 ± 0.8 kPa vs. PaCO_2_ = 6.9 ± 1.7 kPa, *P* < 0.05). There was good agreement in PeCO_2_ calculated with DBag vs. VCap (post-cardiac surgery bias = 0.04 ± 0.19 kPa; ARDS bias = 0.03 ± 0.27 kPa) and relatively low agreement with DBag vs. InCal (post-cardiac surgery bias = −1.17 ± 0.50 kPa; ARDS mean bias = −0.15 ± 0.53 kPa). These differences strongly affected calculated V_D_/V_T_. For example, in patients with ARDS, V_D_/V_T_calculated with Enghoff-InCal was much higher than Bohr-VCap (V_D_/V_T__Enghoff-InCal_ = 66 ± 10 % vs. V_D_/V_T__Bohr-VCap_ = 45 ± 7 %; *P* < 0.05).

**Conclusions:**

Different techniques to measure PACO_2_ and PeCO_2_ result in clinically relevant mean and individual differences in calculated V_D_/V_T_, particularly in patients with ARDS. Volumetric capnography is a promising technique to calculate true Bohr dead space. Our results demonstrate the challenges clinicians face in interpreting an apparently simple measurement such as V_D_/V_T_.

**Electronic supplementary material:**

The online version of this article (doi:10.1186/s13054-016-1311-8) contains supplementary material, which is available to authorized users.

## Background

Physiological dead space (V_D,phys_) represents the fraction of ventilation not participating in gas exchange, including the airway (or anatomical) dead space (V_D,aw_; i.e., ventilation of the conducting airways) and alveolar dead space (V_D,alv_; i.e., ventilation receiving no pulmonary artery perfusion). In patients with acute respiratory distress syndrome (ARDS), dead space has prognostic value [1–4] and can be used to guide ventilator settings [5–8]. However, dead space is rarely calculated in clinical practice, because assessment of dead space is perceived as challenging and misunderstanding exists on different methods of calculation.

The first method used to calculate dead-space fraction (V_D_/V_T_) was introduced in 1891 by Christian Bohr [9]:

1 $$ \frac{{\mathrm{V}}_{\mathrm{D}}}{{\mathrm{V}}_{\mathrm{T}}}=\frac{{\mathrm{PACO}}_2\hbox{-} {\mathrm{PeCO}}_2}{{\mathrm{PACO}}_2} $$

where V_D_ is dead-space volume (i.e., volume not participating in gas exchange), V_T_ is total exhaled volume, PACO_2_ is the partial pressure of carbon dioxide in alveolar air, and PeCO_2_ is the partial pressure of carbon dioxide in mixed expired air. V_D_ calculated using Bohr’s equation accurately measures V_D,phys_ [10]. However, difficulties with measurement of PACO_2_ led to rejection of this method. In 1938, Enghoff proposed replacement of PACO_2_ by partial pressure of carbon dioxide in arterial blood (PaCO_2_), also known as the Enghoff modification [11]. This modification is in general use today, but it comes with limitations. By substituting PaCO_2_ for PACO_2_, intrapulmonary shunt and diffusion limitations are taken into the equation, resulting in a falsely elevated dead-space fraction [10, 12]. Therefore, the Enghoff modification of Bohr’s equation is not a measure of dead space as such but a global index of gas exchange impairment. Nevertheless, in clinical practice, the Enghoff modification is often falsely referred to as V_D,phys_. Another modification of the traditional Bohr formula uses the end-tidal partial pressure of carbon dioxide (PETCO_2_) instead of PACO_2_ [13]. In healthy subjects at rest, PETCO_2_ almost equals PaCO_2_ (and PACO_2_), but during heavy exercise PETCO_2_ overestimates PaCO_2_ and in lung disease PETCO_2_ underestimates PaCO_2_ [14–16]. Recently, a novel technique for determining PACO_2_ based on volumetric capnography was developed and validated [17, 18]. With this technique, the eliminated concentration of CO_2_ is plotted against the expired tidal volume, which allows breath-to-breath calculation of PACO_2_ and Bohr dead space. However, in humans, volumetric capnography-based PACO_2_ has been applied only to healthy and anesthetized subjects [19].

In addition to the difficulties with measurement of PACO_2_ in Bohr’s formula, there are different techniques for measuring its second component, PeCO_2_. First, with a Douglas bag, expired air can be collected and analyzed for the fraction of CO_2_. However, this method is labor-intensive, and, in mechanically ventilated patients, gas compression and ventilator bias flow dilute expired air and should be corrected for [20]. Second, indirect calorimetry measures CO_2_ production $\left({\overset{.}{\mathrm{V}}\mathrm{C}\mathrm{O}}_2\right)$, which can be used to calculate PeCO_2_. Third, the most commonly used and easiest method to determine PeCO_2_ is volumetric capnography.

The purpose of the present study was to evaluate how different techniques of measuring PACO_2_ and PeCO_2_ affect calculated dead-space ventilation in mechanically ventilated patients with ARDS and normal lung function. PACO_2_ was calculated using volumetric capnography or replaced with PaCO_2_. PeCO_2_ was calculated using the Douglas bag, indirect calorimetry, and volumetric capnography.

## Methods

### Study subjects

We conducted a prospective, observational study in the intensive care unit of the Radboud University Medical Center in Nijmegen, The Netherlands. The protocol was approved by the institutional review board (CMO regio Arnhem-Nijmegen) and was in accordance with the ethical standards laid down in the 1964 Declaration of Helsinki and its later amendments. The institutional review board waived the need for informed consent.

### Study design

Two patient groups were studied: 15 patients who underwent elective post-cardiac surgery and 15 patients fulfilling the Berlin Definition of ARDS [21]. Exclusion criteria were hemodynamic instability (mean arterial pressure <65 mmHg despite vasopressors) in both groups and past medical history of lung disease in the post-cardiac surgery patients. All patients were ventilated with a SERVO-i ventilator (Maquet Critical Care, Sölna, Sweden) and disposable tubing (patients with ARDS, Evaqua breathing circuit, Fisher & Paykel Healthcare, Auckland, New Zealand; post-cardiac surgery patients, Limb-O breathing circuit, GE Healthcare, Little Chalfont, UK). Mechanical ventilator settings were not adjusted during the study. Fraction of inspired oxygen (FiO_2_) and positive end-expiratory pressure (PEEP) were set according to the lower PEEP/higher FiO_2_ arm of the ARDSNet protocol.

### Calculating dead-space ventilation

V_D_/V_T_ was calculated simultaneously using four methods: (1) Enghoff-Douglas bag (DBag), (2) Enghoff-indirect calorimetry (InCal), (3) Bohr-volumetric capnography (VCap), and (4) Enghoff-VCap. All measurements were performed within the same 5 minutes to ensure that methods could be accurately compared.

#### Enghoff-DBag

Dead space with Enghoff-DBag was calculated using the Enghoff modification:

2 $$ \frac{{\mathrm{V}}_{\mathrm{D}}}{{\mathrm{V}}_{\mathrm{T}}}=\frac{{\mathrm{PaCO}}_2\hbox{-} {\mathrm{PeCO}}_2}{{\mathrm{PaCO}}_2} $$

PaCO_2_ was determined using an arterial blood gas sample derived from an arterial catheter. Expired air was collected during 2 to 3 minutes to obtain a representative sample from the expiratory port of the ventilator in a 25-L Douglas bag. PeCO_2_ was determined using a sample taken from the bag with a 50-ml syringe (BD Plastipak; BD, Drogheda, Ireland), which was analyzed using the Siemens Rapidlab 865 (Diamond Diagnostics, Holliston, MA, USA). The coefficient of repeatability of the Rapidlab was 0.03 kPa.

PeCO_2_ in the expired air was corrected for dilution due to gas compression in the ventilator circuit [20], as well as for ventilator bias flow (2 L/min):

3 $$ \mathrm{compression}\ \mathrm{volume} = \mathrm{circuit}\ \mathrm{compliance} \times \left({\mathrm{P}}_{\mathrm{peak}} - \mathrm{PEEP}\right) $$

4 $$ \mathrm{bias}\ \mathrm{flow}\ \mathrm{volume} = \mathrm{expiratory}\ \mathrm{time} \times \mathrm{bias}\ \mathrm{flow} $$

5 $$ \mathrm{corrected}\ {\mathrm{PeCO}}_2 = {\mathrm{PeCO}}_2 \times \left(\frac{{\mathrm{V}}_{\mathrm{T}}}{{\mathrm{V}}_{\mathrm{T}}\ \hbox{-}\ \left(\mathrm{compression}\ \mathrm{volume} + \mathrm{bias}\ \mathrm{flow}\ \mathrm{volume}\right)}\right) $$

where P_peak_ is inspiratory peak pressure. The compliance of the ventilator circuit was determined during an internal ventilator test in each patient.

#### Enghoff-InCal

Dead space with Enghoff-InCal was calculated using the Enghoff modification (Eq. 2). PeCO_2_ was derived from indirect calorimetry. Indirect calorimetry was performed with a metabolic analyzer (CARESCAPE Monitor B650; GE Healthcare, Helsinki, Finland) to measure $\overset{.}{\mathrm{V}}{\mathrm{CO}}_2$. Gas sampling was performed via side-stream sampling with a connection piece (dead space 9.5 ml) distal to the Y-piece. PeCO_2_ was calculated as follows:

6 $$ {\mathrm{PeCO}}_2 = \mathrm{k}\times \frac{{\overset{.}{\mathrm{V}}\mathrm{C}\mathrm{O}}_2}{\overset{.}{\mathrm{V}}} $$

where k is the gas constant (0.115 when expressing PeCO_2_ in kilopascals), $\overset{.}{\mathrm{V}}{\mathrm{CO}}_2$ is CO_2_ production (in milliliters per minute standard temperature dry pressure] and $\overset{.}{\mathrm{V}}$ is minute ventilation (in liters per minute body temperature standard pressure). $\overset{.}{\mathrm{V}}{\mathrm{CO}}_2$ and $\overset{.}{\mathrm{V}}$ were stored per minute on the monitor. An average of at least 5 minutes was used for calculations.

#### Bohr-VCap

For Bohr-VCap, dead space was calculated using the Bohr equation (Eq. 1). Flow and arterial carbon dioxide tension (PCO_2_) were measured using the NICO capnograph (Philips Respironics, Murrysville, PA, USA). The capnograph consists of a mainstream CO_2_ sensor (CAPNOSTAT; Philips Respironics) using infrared absorption technology and a flow sensor connected to the CAPNOSTAT attached distal to the Y-piece (dead space 8.5 ml). Flow and PCO_2_ were acquired at a sampling rate of 200 Hz and stored for offline analysis.

Offline analysis was performed with an algorithm developed for MATLAB (MathWorks, Natick, MA, USA). The volumetric capnogram was obtained per breath by plotting PCO_2_ against expired volume. The volumetric capnogram was averaged over a period of 2 minutes, selected by visual inspection to ensure no artifacts. The latter was necessary to correct for respiratory variability (particularly with pressure support ventilation) and thus obtain a representative breath (Additional file 1: Fig. S1). PACO_2_, PeCO_2_, and V_D,aw_ were determined from the volumetric capnogram using model fitting (Additional file 1: Fig. S2) as described by Tusman and colleagues [17]. Briefly, mean PACO_2_ was calculated as the midpoint of phase III in the volumetric capnogram, and PeCO_2_ was calculated as the area under the curve of the volumetric capnogram divided by expiratory volume. The position of the airway-alveolar interface (V_D,aw_) was calculated as the inflection point of phase II of the volumetric capnogram*.* Consequently, V_D,alv_ could be calculated as follows:

7 $$ {\mathrm{V}}_{\mathrm{D},\mathrm{a}\mathrm{l}\mathrm{v}} = {\mathrm{V}}_{\mathrm{D},\mathrm{phys}}-{\mathrm{V}}_{\mathrm{D},\mathrm{a}\mathrm{w}} $$

#### Enghoff-VCap

For Enghoff-VCap, dead space was calculated using the Enghoff modification (Eq. 2). PeCO_2_ was determined from the volumetric capnogram as described in the preceding subsection.

### Statistical analysis

Statistical analysis was performed with Prism 5 software (GraphPad Software Inc., San Diego, CA, USA). The normality of the distribution of the data was determined with the D’Agostino-Pearson test. Normally distributed variables were expressed as mean ± standard deviation. Nonparametric data were expressed as median [interquartile range]. Paired *t* tests and Bland-Altman analysis were used for comparisons. *P* < 0.05 was considered statistically significant.

## Results

**Fig. 1:**
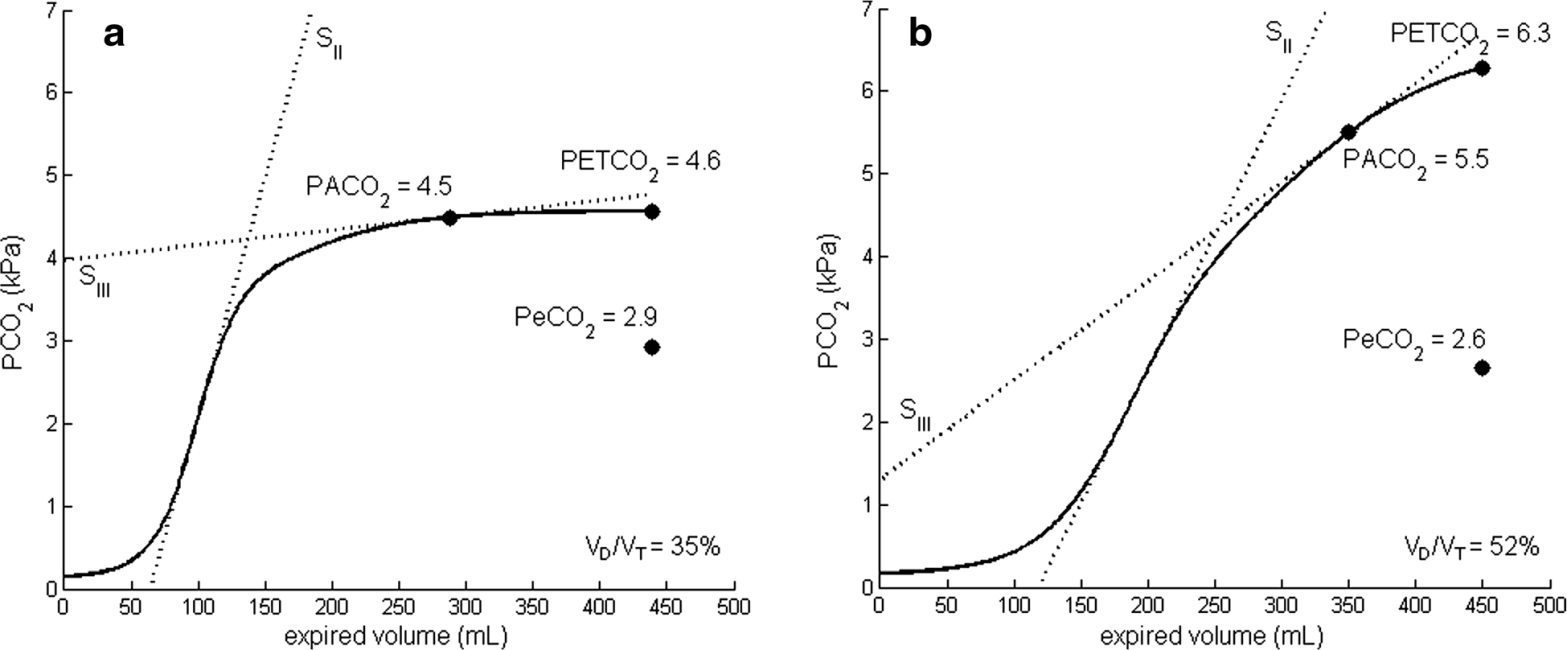
Representative examples of a volumetric capnogram for both patient groups. Volumetric capnogram of a post-cardiac surgery patient (**a**) and a patient with acute respiratory distress syndrome (**b**) with values of PACO_2_, PETCO_2_, PeCO_2_, and dead-space fraction (V_D_/V_T_). S_II_ and S_III_ are the slopes of phases II and III, respectively, of the volumetric capnogram (see Additional file 1: Fig. S2). *PaCO*_*2*_ partial pressure of carbon dioxide in arterial blood, *PACO*_*2*_ partial pressure of carbon dioxide in alveolar air, *PeCO*_*2*_ partial pressure of carbon dioxide in mixed expired air, *PETCO*_*2*_end-tidal partial pressure of carbon dioxide

Table 1 reports patient characteristics and ventilator settings. Figure 1 shows representative examples of the volumetric capnogram of post-cardiac surgery patients and patients with ARDS. Average values of PACO_2_, PaCO_2_, PeCO_2_, and V_D_/V_T_ for both groups, measured and calculated with the different methods, are given in Table 2.

**Table 1:** Patient characteristics and ventilator settings

	Post-cardiac surgery (*n* = 15)	ARDS (*n* = 15)
Age, years	71 ± 11	56 ± 17
Gender, F/M	6/9	3/12
Weight, kg	80 ± 14	80 ± 21
Height, cm	172 ± 9	178 ± 10
Admission diagnosis	11 CABG	14 pneumonia
	4 valve surgery	1 abdominal sepsis
Pulmonary comorbidities	None	1 asthma
		1 interstitial lung disease
		1 lung cancer
PaO_2_/FiO_2_, mmHg	354 ± 76	153 ± 38
Aa-gradient, mmHg	108 ± 51	245 ± 74
Ventilation mode	15 PRVC	9 assisted ventilation
		6 controlled ventilation
PEEP, cmH_2_O	5 [5–7]	12 [10–14]
Tidal volume, ml/kg PBW	8.3 ± 0.9	6.8 ± 1.2
Time on ventilator	1.8 ± 0.8 h	11.5 ± 11.4 days

*Aa-gradient* alveolar-arterial oxygen concentration gradient, *ARDS* acute respiratory distress syndrome, *CABG* coronary artery bypass graft, *FiO*_*2*_ fraction of inspired oxygen, *PaO*_*2*_ partial pressure of oxygen in arterial blood, *PBW* predicted body weight, *PEEP* positive end expiratory pressure, *PRVC* pressure-regulated volume control

Data are presented as mean ± SD or median [IQR]

**Table 2:** Dead space and its parameters in post-cardiac surgery patients and patients with acute respiratory distress syndrome calculated with different methods

	Post-cardiac surgery (*n* = 15)				ARDS (*n* = 15)			
	Enghoff-DBag	Enghoff-InCal	Bohr-VCap	Enghoff-VCap	Enghoff-DBag	Enghoff-InCal	Bohr-VCap	Enghoff-VCap
PACO_2_, kPa	–	–	4.3 ± 0.6	–	–	–	3.9 ± 0.8	
PaCO_2_, kPa	5.2 ± 0.5	5.2 ± 0.5	–	5.2 ± 0.5	6.9 ± 1.7	6.9 ± 1.7	–	6.9 ± 1.7
PeCO_2_, kPa	2.7 ± 0.2	3.8 ± 0.5^a^	2.6 ± 0.4	2.6 ± 0.4	2.2 ± 0.4	2.3 ± 0.7	2.1 ± 0.5	2.1 ± 0.5
V_D_/V_T_, %	49 ± 4	26 ± 9^b^	38 ± 5^c^	50 ± 4	67 ± 9	66 ± 10	45 ± 7^d^	68 ± 9

*PACO*_*2*_ mean alveolar carbon dioxide tension, *PaCO*_*2*_ arterial carbon dioxide tension, *PeCO*_*2*_ mixed expired carbon dioxide tension, *V*_*D*_ */V*_*T*_ dead-space fraction, *DBag* Douglas bag, *InCal* indirect calorimetry, *VCap* volumetric capnography

Within-group testing: *P* < 0.05 for ^a^Enghoff-InCal vs. Enghoff-DBag, Bohr-VCap, Enghoff-VCap; ^b^Bohr-VCap vs. Enghoff-DBag, Enghoff-InCal, Enghoff-VCap; ^c^Enghoff-InCal vs. Enghoff-DBag, Bohr-VCap, Enghoff-VCap; ^d^Bohr-VCap vs. Enghoff-DBag, Enghoff-InCal, Enghoff-VCap

### PACO_2_, PETCO_2_, and PaCO_2_

**Fig. 2:**
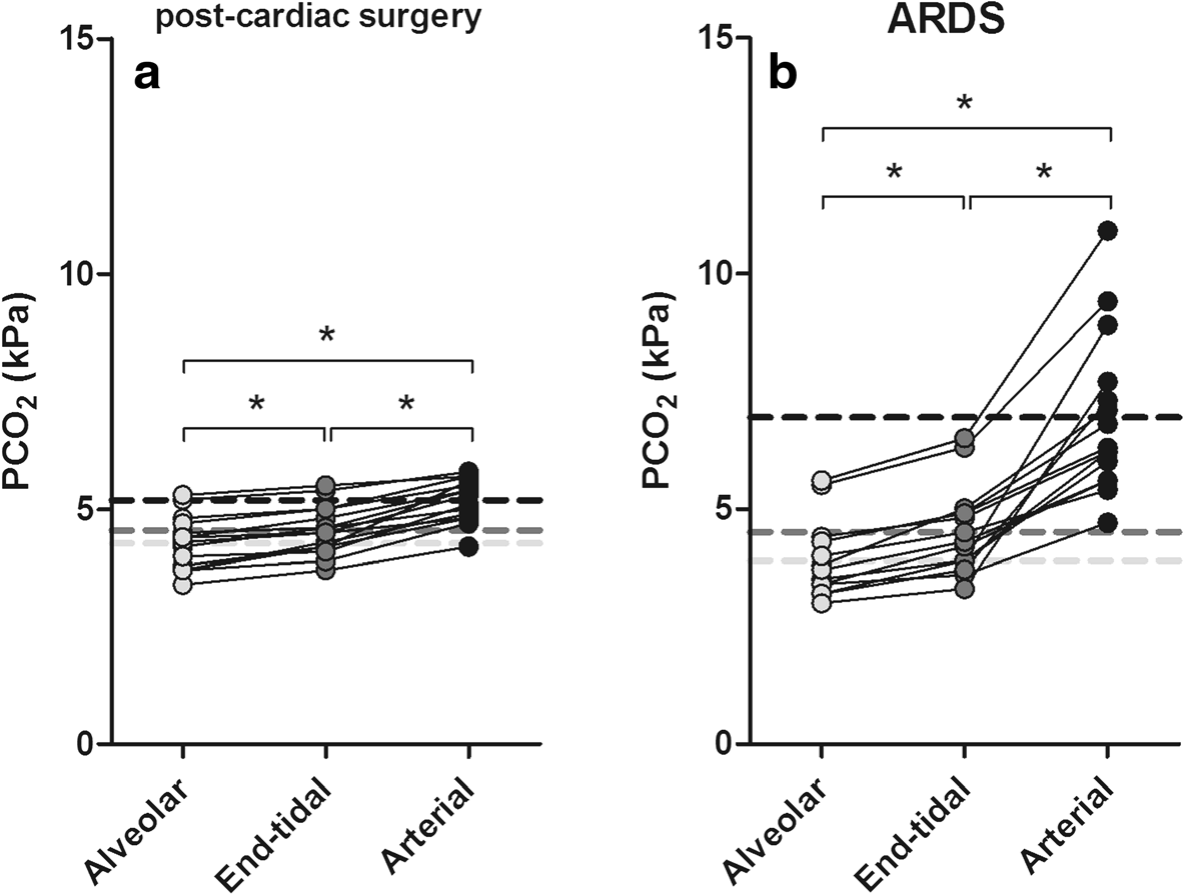
Values of PACO_2_, PETCO_2_, and PaCO_2_ for both patient groups. Individual alveolar, end-tidal, and arterial carbon dioxide tensions in post-cardiac surgery patients (**a**) and patients with acute respiratory distress syndrome (ARDS) (**b**). Alveolar and end-tidal PCO_2_ were obtained with volumetric capnography. The *dashed lines* represent mean values of the parameters with the corresponding colors. *P < 0.05. *PCO*_*2*_ arterial carbon dioxide tension, *PaCO*_*2*_ partial pressure of carbon dioxide in arterial blood, *PACO*_*2*_ partial pressure of carbon dioxide in alveolar air, *PETCO*_*2*_end-tidal partial pressure of carbon dioxide

For both patient groups, there was a significant difference between PACO_2_, PETCO_2_, and PaCO_2_, confirming that these parameters are not interchangeable (Fig. 2). As expected, these differences were much more pronounced in patients with ARDS (Table 2 and Fig. 2). In post-cardiac surgery patients, PETCO_2_ and PaCO_2_ were, respectively, 7 ± 5 % and 23 ± 11 % higher than PACO_2_ vs. 16 ± 7 % and 81 ± 43 % in patients with ARDS.

### PeCO_2_

**Fig. 3:**
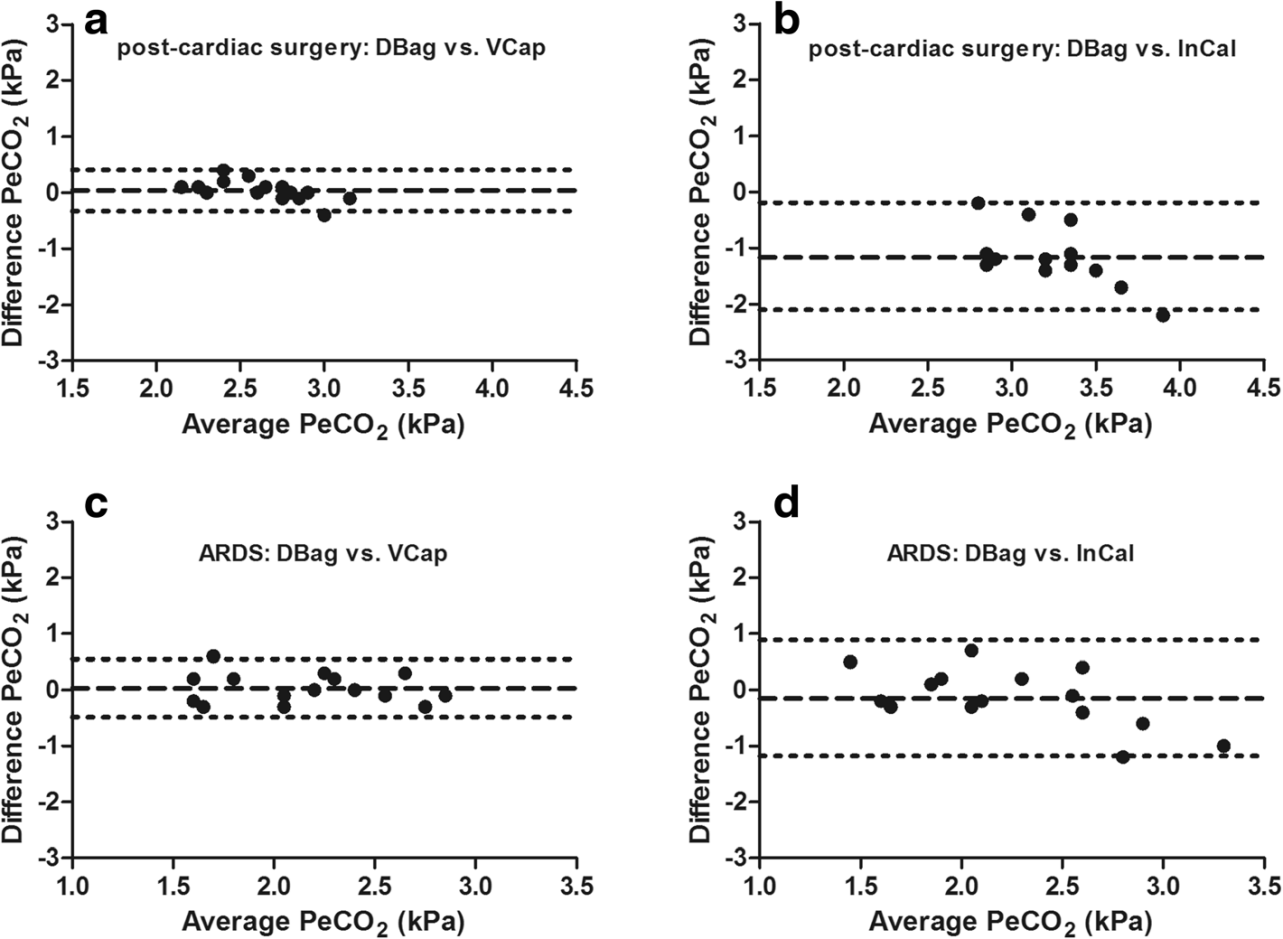
Agreement between different techniques to calculate mixed expired carbon dioxide. Bland-Altman plots comparing mixed expired carbon dioxide (PeCO_2_) calculated by measurements from Douglas bag (DBag) vs. volumetric capnography (VCap) and indirect calorimetry (InCal) in post-cardiac surgery patients (**a** and **b**) and patients with acute respiratory distress syndrome (ARDS) (**c** and **d**). *Dotted lines* represent 95 % limits of agreement, and dashed lines represent mean bias

PeCO_2_ measured with InCal was higher than with DBag and VCap in post-cardiac surgery patients (Table 2). Figure 3 shows Bland-Altman plots of PeCO_2_ measured with DBag vs. InCal and VCap. In post-cardiac surgery patients, the agreement in PeCO_2_ between DBag and VCap was high (mean bias 0.04 ± 0.19 kPa), while the agreement between DBag and InCal was low (mean bias −1.17 ± 0.50 kPa). In patients with ARDS, the agreement in PeCO_2_ between DBag and VCap (mean bias 0.03 ± 0.27 kPa) was comparable to that of post-cardiac surgery patients, but between DBag and InCal (mean bias −0.15 ± 0.53 kPa) it was better than with post-cardiac surgery patients.

DBag vs. VCap had high agreement only if PeCO_2_obtained with DBag was corrected for dilution due to ventilator bias flow and compressible volume (Additional file 1: Fig. S3). PeCO_2_ after correction for bias flow and compressible volume was 0.65 ± 0.11 kPa and 0.39 ± 0.16 kPa higher than when uncorrected for post-cardiac surgery and patients with ARDS, respectively (Additional file 1: Fig. S4).

### Dead space

**Fig. 4:**
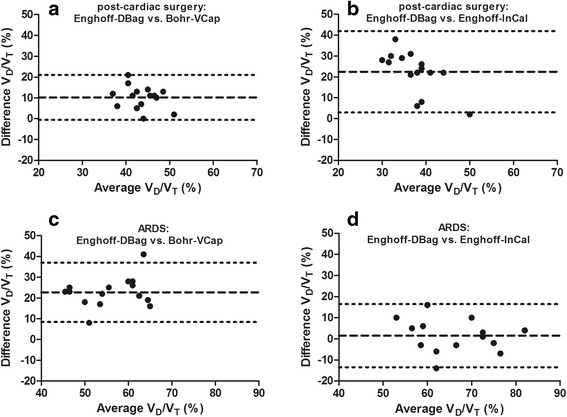
Agreement between different techniques to calculate the dead-space fraction. Bland-Altman plots comparing dead space fraction (V_D_/V_T_) calculated by measurements from Enghoff-Douglas bag (Enghoff-DBag) vs. Bohr volumetric capnography (Bohr-VCap) and Enghoff-indirect calorimetry (Enghoff-InCal) in post-cardiac surgery patients (**a** and **b**) and patients with acute respiratory distress syndrome (ARDS) (**c** and **d**). *Dotted lines* represent 95 % limits of agreement, and dashed lines represent mean bias

Large differences in calculated dead space were present between the four different methods (Table 2). Compared with Bohr-VCap, dead space calculated with Enghoff-VCap (PACO_2_ replaced with PaCO_2_, but similar PeCO_2_) increased dead space by 31 ± 18 % and 52 ± 15 % for the post-cardiac surgery patients and patients with ARDS, respectively. Figure 4 shows Bland-Altman plots of dead space obtained with different methods. In post-cardiac surgery patients, the mean bias in V_D_/V_T_ between Enghoff-DBag vs. Bohr-VCap was 10 ± 6 %, and between Enghoff-DBag vs. Enghoff-InCal it was 22 ± 10 %. In patients with ARDS, the mean bias in V_D_/V_T_ between Enghoff-DBag vs. Bohr-VCap was 23 ± 7 %, and between Enghoff-DBag vs. Enghoff-InCal it was 2 ± 8 %.

**Fig. 5:**
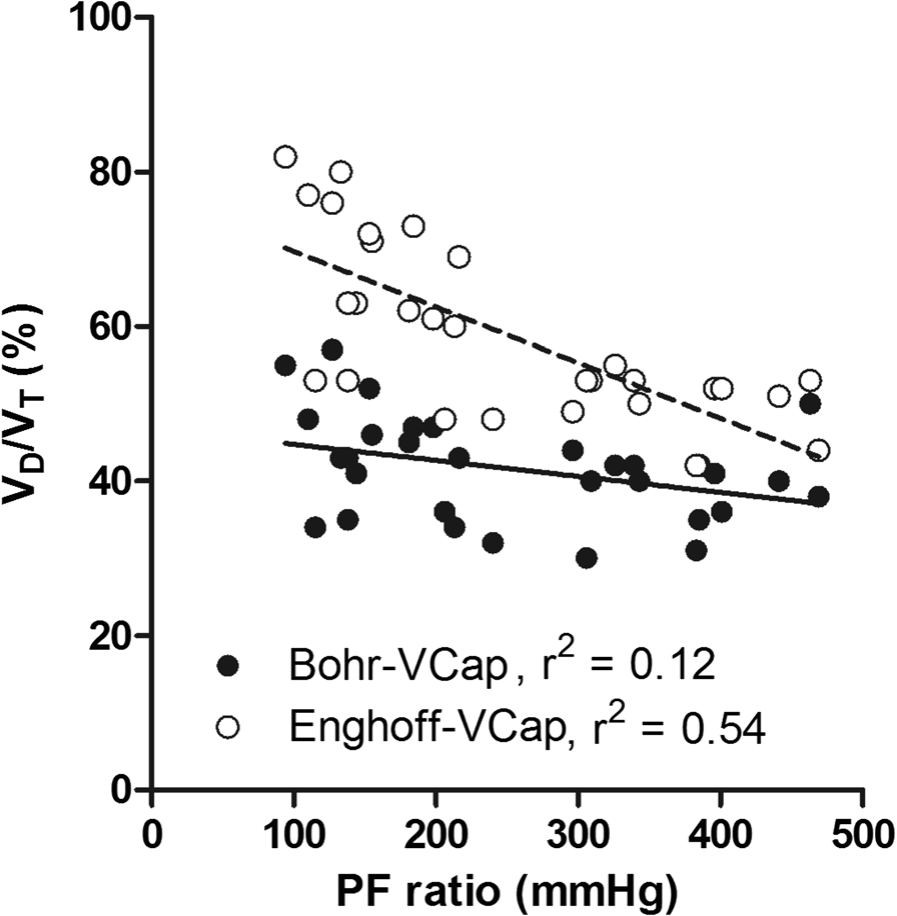
Correlation between dead space and PaO_2_/FiO_2_ ratio. Dead space was calculated using volumetric capnography with PaCO_2_ (Enghoff-VCap) and PACO_2_ (Bohr-VCap). Dead space calculated with Enghoff-VCap shows a strong correlation (*r* ^2^ = 0.54) with PaO_2_/FiO_2_ ratio (PF ratio), whereas this correlation is weak (*r* ^2^ = 0.12) with Bohr-VCap. Thus, the use of PACO_2_ makes dead-space calculation less dependent on intrapulmonary shunts and diffusion impairment. *PaO*_*2*_ partial pressure of oxygen in arterial blood, *PaCO*_*2*_ partial pressure of carbon dioxide in arterial blood, *PACO*_*2*_ partial pressure of carbon dioxide in alveolar air, *PF* PaO_2_/FiO_2_ ratio, *V*_*D*_ */V*_*T*_ dead-space fraction

Changes in intrapulmonary shunt and diffusion have a greater effect on Enghoff-VCap than Bohr-VCap. Partial pressure of oxygen in arterial blood (PaO_2_)/FiO_2_ ratio (PF ratio) may be used as an indicator of these lung parameters. Figure 5 shows the correlation between dead space (Bohr-VCap and Enghoff-VCap) and PF ratio.

Values of V_D,aw_ and V_D,alv_ calculated from the volumetric capnogram are presented and discussed in Additional file 1: Fig. S5.

## Discussion

The present study demonstrates the consequences of applying different techniques for measuring PACO_2_ and PeCO_2_ to calculate dead space in mechanically ventilated patients with ARDS and normal lung function. To our knowledge, we are the first to evaluate a novel method to calculate PACO_2_ using volumetric capnography in patients with ARDS. We show that the differences introduced by replacing PACO_2_ with PaCO_2_ are more pronounced in patients with ARDS than in mechanically ventilated patients with normal lung function. Furthermore, the different techniques used to measure PeCO_2_ introduce potential and clinically relevant sources of error in calculating dead space. These findings have important implications for calculating dead space in daily clinical practice.

### Alveolar and arterial PCO_2_

PACO_2_ is the mean value of CO_2_ in the alveolar compartment, which depends on the balance between perfusion and ventilation of the lung units [10]. The replacement of PACO_2_ with PaCO_2_ in the Bohr formula (Enghoff modification) was proposed to avoid the difficulties of identifying an appropriate PACO_2_. However, in contrast to PACO_2_, PaCO_2_ is affected by intrapulmonary shunt and diffusion impairment [22, 23]. In a healthy lung, the difference between PACO_2_ and PaCO_2_ is minimal but will increase for any gas exchange abnormality. Indeed, we found that the gradient between PACO_2_ and PaCO_2_ is much higher in patients with ARDS than in patients without lung disease (Fig. 2). The former has a strong effect on the calculated dead space in patients with ARDS (52 % increase). Hence, the Enghoff modification of Bohr dead space is not a dead-space measurement as such, but a global index of gas exchange impairment. This is illustrated in Fig. 5, where dead space calculated with Enghoff-VCap shows a strong correlation (*r*^2^ = 0.54) with PF ratio, whereas this correlation is weak (*r*^2^ = 0.12) with Bohr-VCap. In other words, the use of true alveolar PCO_2_ makes dead-space calculation less dependent on intrapulmonary shunt and diffusion impairment. Even with Bohr-VCap, we found that dead space in patients with ARDS was higher than in post-cardiac surgery patients. This may be explained by the difference in lung condition but also by the difference in tidal volume between the groups. A lower tidal volume relatively increases dead space.

### Techniques to measure mixed expired PCO_2_

In the present study, we used three techniques (DBag, VCap, and InCal) to measure PeCO_2_. In the last decade, researchers in several clinical studies compared these techniques as well [24–26]. None of the studies included comparisons of all three techniques, but a high agreement in PeCO_2_ was found previously between VCap and DBag [25] and between VCap and InCal [24, 26]. In accordance with these results, we found a high agreement between DBag and VCap in patients with ARDS and in post-cardiac surgery patients (Fig. 3). However, the accuracy of indirect calorimetry to measure PeCO_2_ appeared lower. First, the 95 % limits of agreement were larger with DBag vs. InCal compared with DBag vs. VCap in both patient groups. Second, the mean bias between DBag and InCal showed large offset in post-cardiac surgery patients.

It is important to note that, with the Douglas bag, expired air is collected at the expiratory limb of the ventilator circuit and is consequently diluted by CO_2_-free air coming from compressed volume and bias flow volume. In the present study, dilution of expired air lowered PeCO_2_. The effect of dilution becomes larger as the ratio between bias flow volume and expired volume increases. This ratio is higher in post-cardiac surgery patients, who have, in general, a relatively long expiration time compared with patients with ARDS. The reliability of using a correction factor to estimate the degree of dilution depends primarily upon the accuracy of the recorded peak pressure and expired tidal volume for compressed volume [24] and the expiratory time for bias flow volume. Volumetric capnography measures expired CO_2_ distal to the Y-piece of the ventilator circuit and is unaffected by compression volume and bias flow.

### Clinical implications

The techniques used in the present study cause large differences in calculated dead space (Table 2 and Fig. 4) and demonstrate the difficulties encountered in clinical practice. These differences are dependent on the choice of dead-space formula (Bohr or Enghoff modification) and the technique used to measure PeCO_2_, as discussed above, and they have important clinical implications. First, one should never use different techniques to calculate dead space in follow-up of a patient. Second, several studies have demonstrated that elevated dead space in patients with ARDS is associated with an increased risk of mortality [1–4]. The researchers in these studies calculated the Enghoff modification of Bohr dead space and thus calculated an index of global gas exchange impairment and not true dead space. Therefore, it is unknown whether true Bohr dead space measured with VCap has similar prognostic value. Third, a question remains regarding which method clinicians should use at the bedside to determine dead space. The answer depends on the clinical problem to be addressed and the techniques available. Nowadays, there are several capnographs available that provide dead-space values at the bedside. These include stand-alone monitors (e.g., NICO capnograph) or modules incorporated into the mechanical ventilator (e.g., Evita Infinity V500, Dräger Medical, Lübeck, Germany; HAMILTON-G5, Hamilton Medical, Bonaduz, Switzerland). However, these capnographs are not able to calculate alveolar PCO_2_ (and thus Bohr dead space), as demonstrated in our study, and still require manual entry of PaCO_2_ to determine dead space according Enghoff’s modification. If one’s goal is to improve or follow up overall gas exchange, it complies is appropriate to take an arterial blood gas samples and use the Enghoff modification. However, if one wants to evaluate the effect of different ventilator settings on alveolar dead space, one must calculate Bohr dead space (i.e., physiological dead space). For example, differences in end-expiratory lung volume and extrinsic PEEP levels greatly affect airway and alveolar dead space [27–29]. In case of high PEEP, vessels can be compressed by overdistention of alveoli, which causes alveolar perfusion to decrease and consequently increases alveolar dead space. However, high PEEP may also overcome atelectasis and thereby increase alveolar recruitment and reduce pulmonary shunting. If dead space is measured using the Enghoff modification, it is not possible to discriminate between the effects of PEEP on pulmonary shunt and alveolar dead space.

### Study limitations

The gold standard for calculating Bohr dead space is the mathematical algorithm of the multiple inert gas elimination technique (MIGET), an approach that allows quantification of all the pulmonary and extrapulmonary determinants of arterial oxygenation. Due to the complexity of the MIGET technique, it is never used in clinical practice and rarely in clinical studies. Nevertheless, it is reasonable to assume that Bohr dead space calculated using volumetric capnography in our study provided an accurate estimate. First, the concept of obtaining PACO_2_ from the midportion of phase III with volumetric capnography has recently been validated against the MIGET technique in lung-lavaged pigs [18]. Second, our values of dead space were comparable with the only clinical study in patients with ARDS in the current era of low tidal volumes in which researchers calculated dead space using both the MIGET technique and the Enghoff modification [30]. In that study, V_D_/V_T,Bohr_ was 40 % and V_D_/V_T,Enghoff_ was 65 %, compared with 45 % and 68 %, respectively, in our present study.

Previously, using the similar volumetric capnography technique as used in the present study, V_D_/V_T,Bohr_ was found to be 23 % in healthy subjects and 28 % in anesthetized patients undergoing elective, noncomplex, and neither laparoscopic nor thoracic surgeries in supine position [19]. In our post-cardiac surgery patients, V_D_/V_T,Bohr_ was markedly higher at 38 %. This difference is most likely the result of a longer surgical procedure, open chest surgery, hypovolemia, and higher PEEP in our post-cardiac surgery patients.

With volumetric capnography, the calculation of PACO_2_ depends on the determination of the intersections of the tangents of phases II and III (Additional file 1: Fig. S2) [17]. In post-cardiac surgery patients and in most patients with ARDS, this intersection is present. However, in some patients with ARDS, phase III can be very steep due to severe heterogeneity of the lung. Consequently, there is no definite transition from phase II to phase III and hence no intersection of the tangent of phases II and III, which leads to false calculation of PACO_2_. The latter occurred in one of our patients, who was excluded from analysis.

## Conclusions

Use of different techniques to measure PACO_2_ and PeCO_2_ results in clinically relevant mean and individual differences in calculated V_D_/V_T_, particularly in patients with ARDS. Volumetric capnography is a novel and promising technique for calculating true Bohr dead space. Our results demonstrate the complexity of gas exchange in patients with ARDS and the challenges clinicians face in interpreting an apparently simple measurement such as dead space. Awareness of the chosen technique, as well as interpretation and consistent use, is highly important when calculating dead-space ventilation as a prognostic marker or guidance for treatment.

## Key messages

- Different available techniques to measure partial pressure of CO_2_ in alveolar and mixed expired air result in clinically relevant differences in calculated V_D_/V_T_, particularly in patients with ARDS.
- Volumetric capnography is a novel and promising technique for calculating true Bohr dead space.
- Awareness of the chosen technique, as well as interpretation and consistent use, are highly important when calculating dead-space ventilation as a prognostic marker or guidance for ventilator settings.

## Additional file

Additional file 1: Figures depicting volumetric capnography, corrections for dead space analysis with the Douglas bag, and the different components of dead space. (DOCX 1371 kb)

## Abbreviations

Aa-gradient: alveolar-arterial oxygen concentration gradient
ARDS: acute respiratory distress syndrome
CABG: coronary artery bypass graft
DBag: Douglas bag
FiO_2_: fraction of inspired oxygen
InCal: indirect calorimetry
MIGET: multiple inert gas elimination technique
PaCO_2_: partial pressure of carbon dioxide in arterial blood
PACO_2_: partial pressure of carbon dioxide in alveolar air
PaO_2_: partial pressure of oxygen in arterial blood
PBW: predicted body weight
PCO_2_: arterial carbon dioxide tension
PeCO_2_: partial pressure of carbon dioxide in mixed expired air
PEEP: positive end-expiratory pressure
PETCO_2_: end-tidal partial pressure of carbon dioxide
PF: PaO_2_/FiO_2_ ratio
P_peak_: inspiratory peak pressure
PRVC: pressure-regulated volume control
VCap: volumetric capnography
V_D,alv_: alveolar dead space
V_D,aw_: airway dead space
V_D,phys_: physiological dead space
V_D_/V_T_: dead-space fraction
$$ \overset{.}{\mathrm{V}} $$: minute ventilation
$\overset{.}{\mathrm{V}}{\mathrm{CO}}_2$: carbon dioxide production per minute
