# Supplementary material for: Assessment of dead-space ventilation in patients with acute respiratory distress syndrome: a prospective observational study
Source: Crit Care. 2016 May 5;20:121. doi: 10.1186/s13054-016-1311-8 (PMC4857382; doi:10.1186/s13054-016-1311-8)
Supplement: Additional file 1: — Figures depicting volumetric capnography, corrections for dead space analysis with the Douglas bag, and the different components of dead space. (DOCX 1371 kb) [file 13054_2016_1311_MOESM1_ESM.docx]

**Assessment of dead space ventilation in patients with acute respiratory distress syndrome: a prospective observational study**

*Additional figures*

Jonne Doorduin MSc^1^, Joeke L Nollet MSc^1^, Manon PAJ Vugts MD MSc^1^, Lisanne H Roesthuis MSc^1^, Ferdi Akankan^2^, Johannes G van der Hoeven MD PhD^1^, Hieronymus WH van Hees PhD^2^, Leo MA Heunks MD PhD^1^

^1^ Department of Critical Care Medicine and ^2^ Pulmonary Diseases, Radboud university medical center, The Netherlands

**FIGURES**

**Figure S1**

**
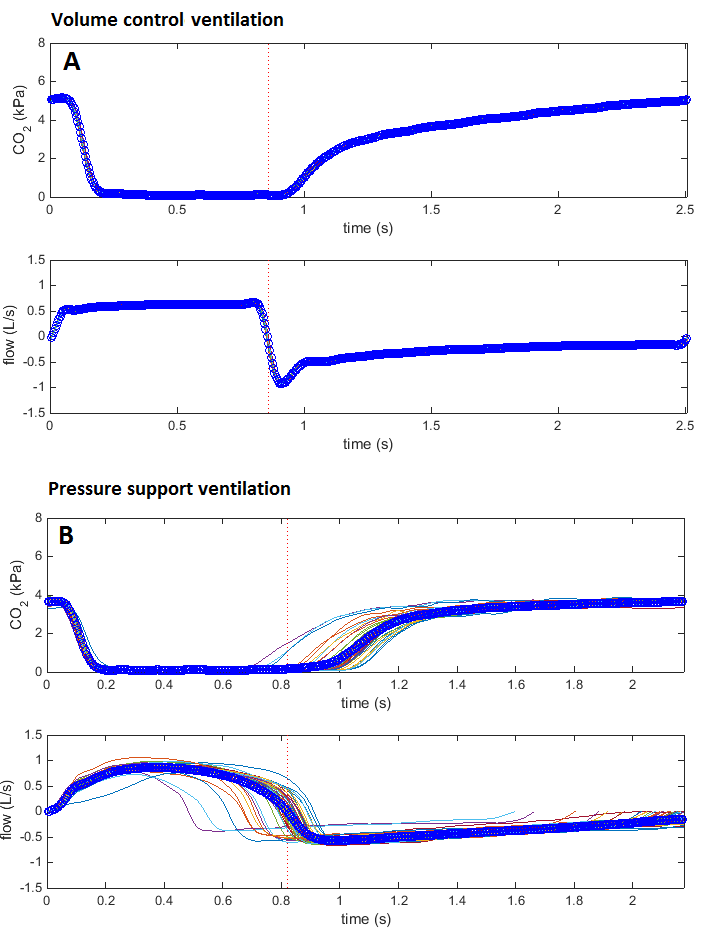
**

*Calculation of average breath for VCap method*

To obtain a representative breath to calculate dead space with VCap an average breath (blue circled line) was calculated over a period of at least 2 minutes. With volume control ventilation (panel A) there is almost no respiratory variability and therefore the average breath is plotted exactly over the individual breaths (n=49). With pressure support ventilation (panel B) there is a certain degree of respiratory variability, as illustrated by the plotted individual breaths (n=56).

**Figure S2**


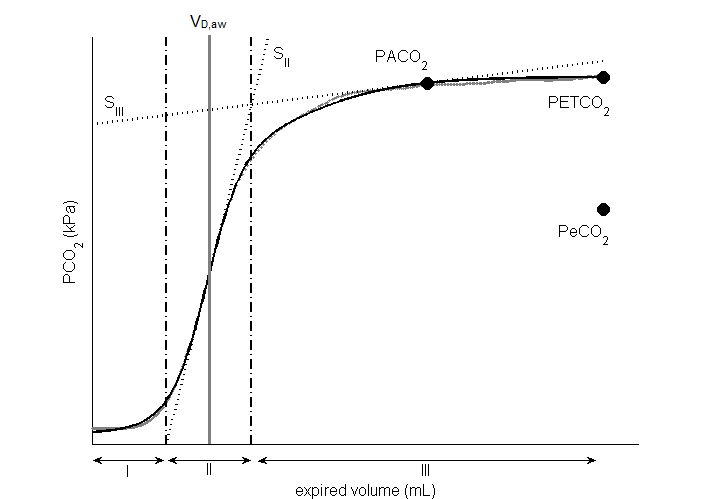


*Example of volumetric capnogram with description of derived variables.*

Gray curve (largely under black curve) represent original data from multiple averaged breaths, and the black curve the mathematical model fit. PeCO_2_ was calculated as area under the curve divided by the expiratory volume. Phase I, II, and III represent, respectively, the portion of expired volume free of CO_2_, CO_2_ originating from lung units with different ventilatory and perfusion rates, and pure alveolar gas. Phase II extends from the point of maximum rate of change of the second derivative of the curve to the intersection of S_II_ and S_III_. S_II_ and S_III_ are the slopes of phase II and III, respectively. PACO_2_ has been defined as the midpoint of phase III. V_D,aw_ (gray vertical line) was calculated as the inflection point of phase II. A more detailed description of the model fit and its calculations can be found in the work of Tusman and colleagues [E1].

**Figure S3**


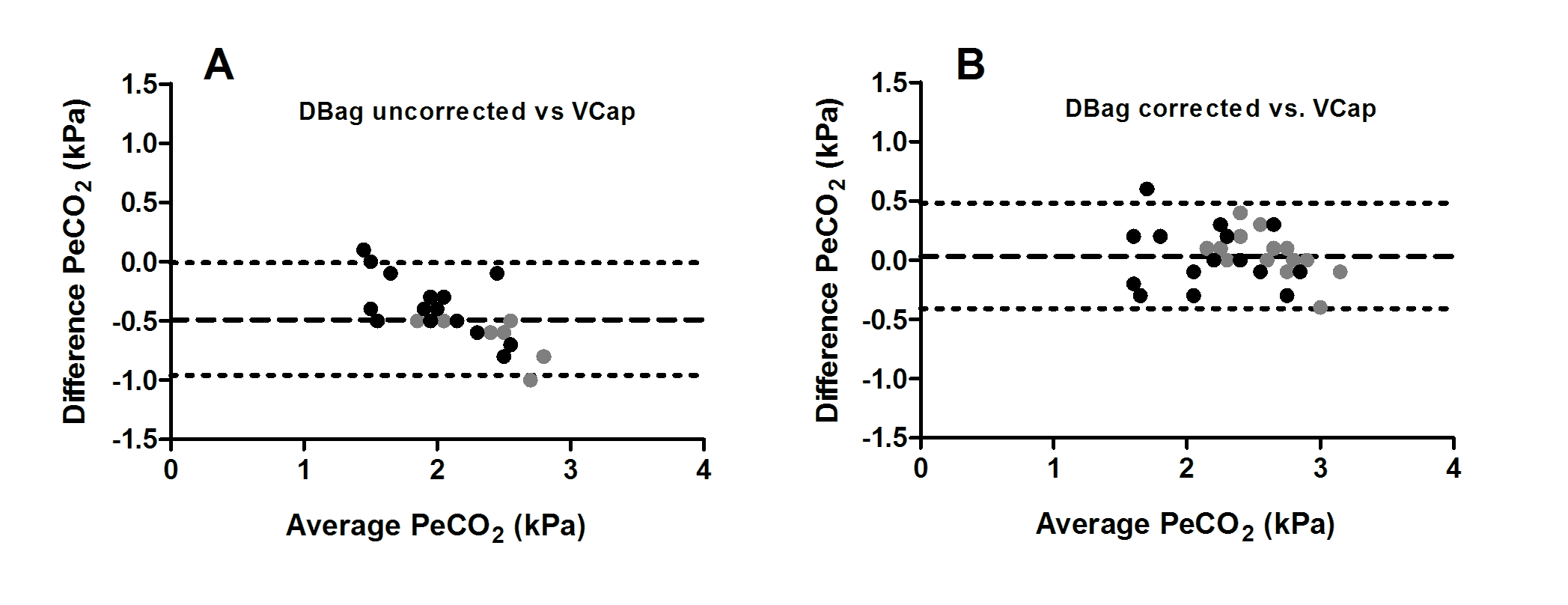


*Agreement between DBag and VCap to calculate PeCO_2_ before and after correction for bias flow and compressible volume.*

Bland-Altman plots comparing mixed expired CO_2_ (PeCO_2_) calculated by measurements from Douglas Bag (DBag) uncorrected vs. volumetric capnography (VCap) (A), and DBag corrected vs. VCap (B) in all patients. Gray dots represent post-cardiac surgery patients and black dots ARDS patients. Dotted lines represent 95% limits of agreement and striped line is mean bias.

**Figure S4**


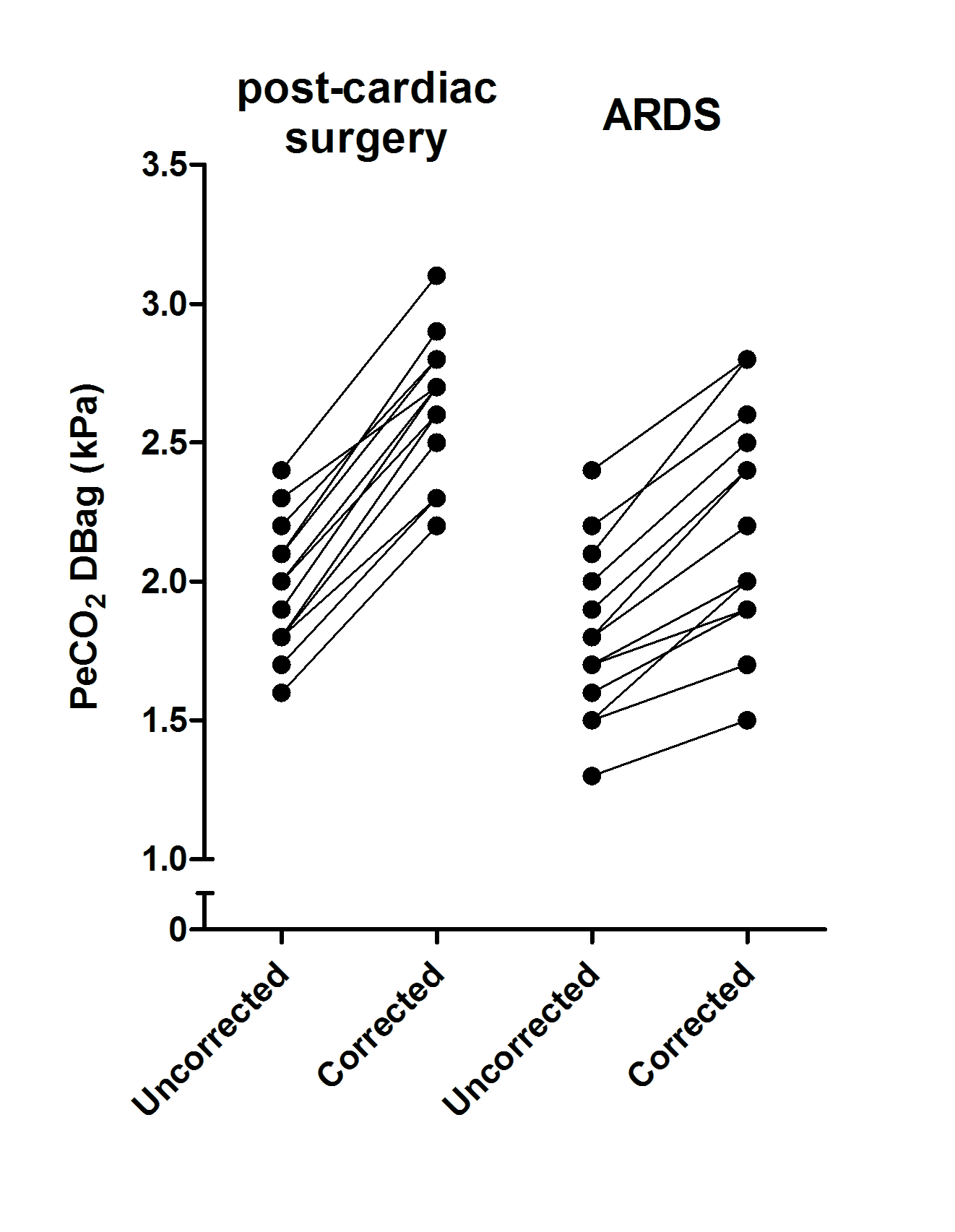


*Effect of* *bias flow and compressible volume correction on PeCO_2_.*

Mixed expired PCO_2_ (PeCO_2_) measured with the Douglas bag (DBag) increases after correction for dilution due to bias flow and compressible volume. This effect is larger in post-cardiac surgery patients because these patients, in general, have a longer expiratory time.

**Figure S5**


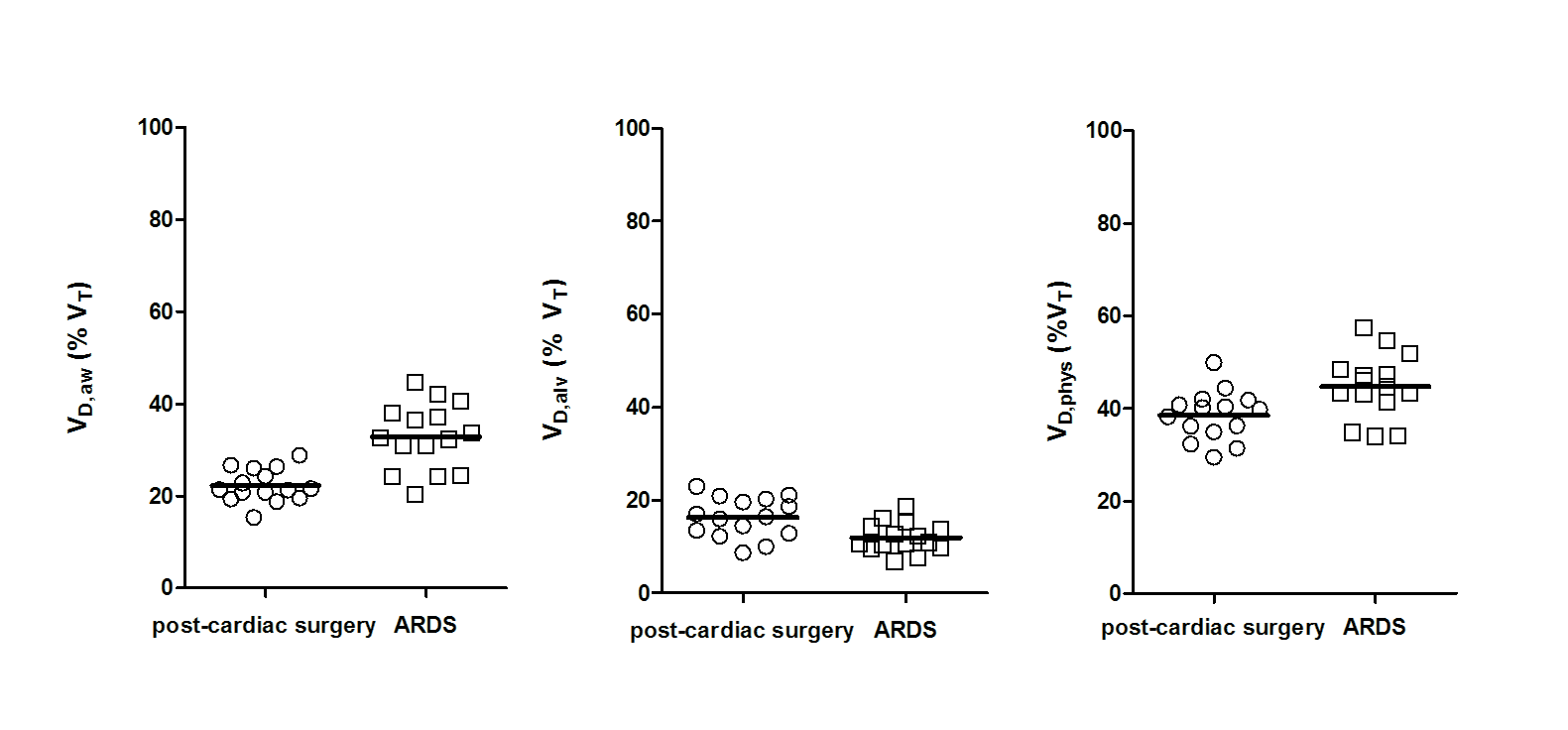


*Airway dead space (V_D,aw_), alveolar dead space (V_D,alv_), and physiological dead space (V_D,phys_) in post-cardiac surgery and ARDS as determined from the volumetric capnogram.*

Recently, reference values were published for V_D,aw_ ,V_D,alv_ , and V_D,Bohr_ using volumetric capnography in anesthetized patients [E2]. Our values of V_D,aw_ (22 %) in the post-cardiac surgery group are comparable to those values (18 %). However, V_D,alv_ (16 %) in our post-cardiac surgery group is higher than V_D,alv_ (9 %) in the anesthetized patients reported in the study by Tusman [E2]. This difference is most likely the result of one or more of the following factors: a longer surgical procedure, open chest surgery, hypovolemia and higher PEEP in our post-cardiac surgery patients. V_D,aw_ (33 %) in ARDS patients is much higher than in the post-cardiac surgery group (p<0.0001). To our knowledge, values of V_D,aw_ in ARDS patients have not been published previously using the current method [E1]. Beydon and colleagues [E3] did measure V_D,aw_ using a different method [E4] in ten ARDS patients and reported a comparable V_D,aw_ of 32 %. Values of V_D,alv_ in the study by Beydon cannot be compared to our data as they used the Enghoff modification for calculation of V_D,phys_. The complex interactions in mechanically ventilated and anesthetized patients make it difficult to give a clear explanation why there is a difference in V_D,aw_ and V_D,alv_ between post-cardiac surgery and ARDS patients. For example, differences in end-expiratory lung volume and PEEP levels greatly affect the airway alveolar interface [E3, E5, E6].

**REFERENCES**

E1. Tusman G, Scandurra A, Bohm SH, Suarez-Sipmann F, Clara F: **Model fitting of volumetric capnograms improves calculations of airway dead space and slope of phase III**. *Journal of clinical monitoring and computing* 2009, **23**(4):197-206.

E2. Tusman G, Gogniat E, Bohm SH, Scandurra A, Suarez-Sipmann F, Torroba A, Casella F, Giannasi S, Roman ES: **Reference values for volumetric capnography-derived non-invasive parameters in healthy individuals**. *Journal of clinical monitoring and computing* 2013, **27**(3):281-288.

E3. Beydon L, Uttman L, Rawal R, Jonson B: **Effects of positive end-expiratory pressure on dead space and its partitions in acute lung injury**. *Intensive Care Med* 2002, **28**(9):1239-1245.

E4. Wolff G, Brunner JX: **Series dead space volume assessed as the mean value of a distribution function**. *International journal of clinical monitoring and computing* 1984, **1**(3):177-181.

E5. Schulz A, Schulz H, Heilmann P, Brand P, Heyder J: **Pulmonary dead space and airway dimensions in dogs at different levels of lung inflation**. *Journal of applied physiology* 1994, **76**(5):1896-1902.

E6. Tusman G, Bohm SH, Suarez-Sipmann F, Turchetto E: **Alveolar recruitment improves ventilatory efficiency of the lungs during anesthesia**. *Canadian journal of anaesthesia = Journal canadien d'anesthesie* 2004, **51**(7):723-727.
